# miRNA expression haplotype in Hispanics with endometriosis

**DOI:** 10.3389/frph.2025.1663755

**Published:** 2025-09-25

**Authors:** Flor Daniela Alday-Montañez, Brandon Daniel Lariz-Nevárez, Víctor Josué Carrasco-Urrutia, Daniel Dickens-Terrazas, Adali Barragán-Farías, Gloria Erika Mejía-Carmona, Robert Arthur Kirken, Alfonso Enrique Bencomo-Alvarez, Naún Lobo-Galo, Alejandra Vargas-Caraveo, Ángel Gabriel Díaz-Sánchez, Elisa Robles-Escajeda, Alejandro Martínez-Martínez

**Affiliations:** ^1^ Department of Chemical-Biological Sciences, Autonomous University of Ciudad Juarez, Ciudad Juarez, Chihuahua, Mexico; ^2^ Department of Advanced Gynaecology and Robotic Surgery, Endofem, Ciudad Juarez, Chihuahua, Mexico; ^3^ Department of Pathology, IMSS, Zone Hospital Number 6, Ciudad Juarez, Chihuahua, Mexico; ^4^ Border Biomedical Research Center, College of Science, University of Texas at El Paso, El Paso, TX, United States; ^5^ Department of Host-Microbe Interactions, St. Jude Children’s Research Hospital, Memphis, TN, United States

**Keywords:** translational medicine, pelvic pain, menstruation, dysmenorrhea, dyspareunia, dyschezia, dysuria

## Abstract

**Background:**

Endometriosis affects approximately 10% of women of reproductive age; this prevalence may be underestimated, mostly in developing countries, including Mexican and Hispanic populations, due to socioeconomic barriers and limited access to specialized diagnosis. Although laparoscopy remains the gold standard for diagnosis, highlighting the need for non-invasive biomarkers. Haplotype expression of specific miRNAs acts as a circulating signature in both healthy and disease states, including endometriosis. However, their applicability in Hispanic populations has been unexplored.

**Method:**

This study evaluated the discriminatory capacity of a miRNA expression haplotype in the blood plasma of a Hispanic cohort with laparoscopic confirmed diagnosis (15 patients with endometriosis and 7 from a reference group). The expression levels of miR-451a, miR-3613, miR-125b, let-7b, miR-150, and miR-342 were quantified using qRT-PCR, and their diagnostic performance was assessed through individual ROC curves and multivariate classification models: Logistic regression, CRT, and stacking-based ensemble model.

**Results:**

The miRNA expression haplotype demonstrated high diagnostic accuracy with logistic regression (AUC = 0.914), CRT (AUC = 0.990), and an ensemble model using stacking (AUC = 0.990). Individually, miR-451a (AUC = 0.79), miR-3613 (AUC = 0.714), and let-7b (AUC = 0.667) were the most relevant markers and demonstrated more relevance in the expression haplotype.

**Conclusion:**

These findings suggest that a miRNA-based diagnostic panel could provide a highly sensitive and specific alternative for diagnosing endometriosis in Hispanic populations. However, validation in larger cohorts is essential to confirm reproducibility and assess its clinical utility in different healthcare settings.

## 1 Introduction

Endometriosis is characterized by the ectopic presence of endometrial tissue, associated to chronic inflammation influenced by estrogens and the menstrual cycle (1). It affects 10% of women of reproductive age, accounting for approximately 190 million women worldwide, 30% to 50% of whom experience chronic pelvic pain and infertility (2). Its incidence is underestimated due to the normalization of menstrual pain and the complexity of diagnosis (3). Laparoscopy is both at once: the diagnosis gold standard, as well as treatment, even though it is an invasive technique with multiple operational limitations, including high costs, the need for highly specialized personnel, and its late indication, underscoring the need for a non-invasive, specific, low-cost, and sensitive biomarker (4).

MicroRNAs (miRNAs) have been studied as potential biomarkers for endometriosis, their diagnostic value lies in their stability in systemic circulation and their association with specific pathological metabolic states (5). Unlike other RNAs, miRNAs are protected from degradation through their release in exosomes or their association with proteins such as Argonaute 2 (Ago2), Nucleophosmin 1 (NPM1), and high-density lipoproteins (6). The release of miRNAs into the systemic circulation has been related to pathophysiological processes, such as apoptosis and necrosis in passive secretion, and intercellular communication mediated by exosomes and microvesicles in active secretion (7). The different expression patterns of miRNAs have been associated with various pathologies, including cancers, metabolic disorders, and endometriosis (8).

The diagnostic potential of miRNAs has been found in non-Hispanic populations. A previous study identified a diagnostic signature composed of 109 miRNAs in saliva by next-generation RNA sequencing and a machine learning algorithm, achieving a sensitivity of 96.7% and a specificity of 100% (9). Likewise, a blood-based signature of miRNAs was developed using NGS and artificial intelligence, which showed a sensitivity of 96.8%, a specificity of 100%, and an AUC of 0.984 (10). In addition, a panel of serum miRNAs made it possible to distinguish between different stages of the disease with an AUC curve of 0.940 (8).

Although miRNA expression is intrinsically related to metabolic status, it is a fact that epigenetic factors can cause variations in their expression levels among different ethnic groups, which can affect their performance as “universal” biomarkers and their usefulness in diagnosis in specific groups (11). Therefore, validation of miRNA-specific expression haplotypes for specific populations to diagnose endometriosis is critical (3). Although studies such as those conducted by the National Health and Nutrition Examination Survey (NHANES) report a low prevalence of endometriosis in the Hispanic or Mexican-American population (12, 13) factors such as socioeconomic, cultural, access to health care, and the normalization of menstrual pain, may contribute to an underestimation of its prevalence and incidence in this and other diseases (14, 15). The lack of specific studies on endometriosis in the Hispanic population, with the only relevant work in USA being that of Moustafa et al. (8), highlights the urgent need for specialized research in this population.

The objective of this research was to evaluate a set of six miRNAs in the detection of endometriosis in a Hispanic population in the city of Juarez, México.

## 2 Materials and methods

This research was approved by the Ethics Committee of the Autonomous University of Ciudad Juarez (permit CEI-2023-1-870) and is part of the project entitled “Molecular aspects associated with the recruitment theory of transformed endometrial stem cells in the development of breast cancer”. Various institutions on the border between Ciudad Juarez, Mexico, and El Paso, USA, collaborated, including Hospital Angeles, Star-Medica Hospital, Specialty Medical Center, Lourdes Surgical Center, Autonomous University of Ciudad Juarez, University of Texas at El Paso, Quiescente Molecular, and Endofem.

The miRNAs included in the expression haplotype associated with endometriosis in Hispanic patients were identified through a systematic review of previous studies on differential expression of miRNAs in this disease, with emphasis on their diagnostic potential. The search was carried out in PubMed, Google Scholar, and Scielo databases, using keywords such as “miRNAs”, “microRNAs”, “endometriosis”, “circulating”, “microRNome”, “detection”, and “diagnosis”.

### 2.1 Patients and samples

Based on the methodology proposed by Moustafa et al. (8), an analytical cross-sectional prospective study was conducted, using a double-blinded, non-probabilistic convenience sampling focusing specifically on Hispanic women.

All samples were collected between November 2022 and July 2023 from 8 AM to 10 AM in patients that were fasting for at least 8 h. Prior to venipuncture, each patient signed an informed consent and answered a survey of demographics features. Subsequently, 4 ml of blood was extracted by venipuncture, using tubes with EDTA-K2 (Vacutainer®). The inclusion criteria for the participants were Hispanic women with suspected endometriosis or gynecological pathologies related to chronic pelvic pain, who after blood collection would undergo laparoscopy. The exclusion criteria were patients diagnosed with different forms of gynecologic cancer. Blood was immediately centrifuged at 2,500 rpm for 10 min at room temperature, samples with visible hemolysis were discarded. The plasma was aliquoted and stored at −20°C until analysis.

### 2.2 qRT-PCR

Total RNA, including miRNAs, was extracted from plasma using the miRNeasy Serum/Plasma Advance kit (Cat. No. 217184) following the manufacturer's instructions. The relative quantification of miRNAs was carried out by RT-qPCR using the miRCury LNA RT kit (Cat. No. 339340) for cDNA synthesis. The amplification of the miRNAs was performed with the Open qPCR equipment of CHAI BIO, using the CHAI Green qPCR Master Mix buffer (Cat. No. R02201S). The specific primers for each miRNA were acquired through the GeneGlobeID platform, belonging to the miRCury LNA miRNA PCR Assays kit (Cat. No. 339306, Table 1). The qPCR reactions were prepared according to the proportions described in the manufacturer's protocol. The samples were subjected to a specific thermocycling program for each miRNA, with the small nuclear RNA U6 (YP02119464) used as the reference gene. UniSP6 external quality control (YP00203954) was included in all reactions to ensure the validity of the results. The data obtained were analyzed according to threshold cycles (Ct) and normalized against the reference gene.

**Table 1:** Sequences of miRNAs included in this study.

miRNAs	Sequence	GeneGlobe ID
hsa-miR-125b-5p	UCCCUGAGACCCUAACUUGUGA	YP02119305
hsa-miR-150-5p	UCUCCCAACCCUUGUACCAGUG	YP00205878
hsa-miR-451a	AAACCGUUACCAUUACUGAGUU	YP00204660
hsa-miR-3613-5p	UGUUGUACUUUUUUUUUUGUUC	YP00204516
hsa-miR-342-5p	AGGGGUGCUAUCUGUGAUUGA	YP02119046
hsa-let-7b-5p	UGAGGUAGUAGGUUGUGUGGUU	YP00204124

### 2.3 Data analysis

To evaluate the usefulness of the expression profile of miRNAs in the detection of endometriosis, different statistical analyses were applied, such as ROC curves (Receiver Operating Characteristic), confidence intervals (95% CI) computed via bootstrap resampling (*n* = 1,000), logistic regression, decision algorithms using the CRT (Classification and Regression Trees) method, and the ensemble model using the Stacking technique using the statistical tools of jamovi (16), IBM SPSS (17), and R Studio (18, 19).

## 3 Results

**Figure 1:**
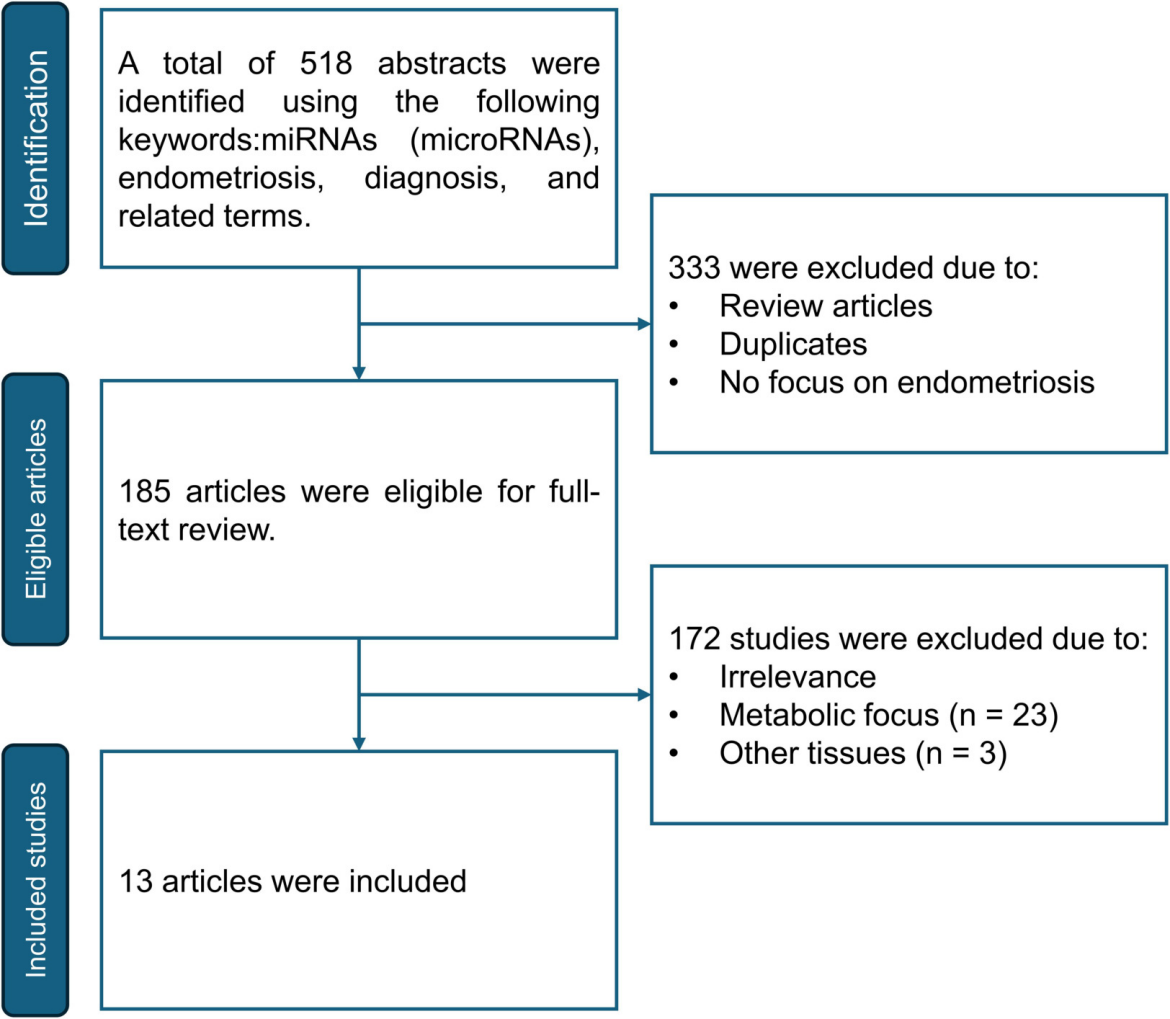
PRISMA diagram of the systematic review carried out for the design of the expression haplotype of miRNAs diagnostic of endometriosis in Hispanic patients.

Based on the systematic review conducted following the PRISMA methodology, a total of 518 abstracts were retrieved, of which 333 were excluded because they were review articles, duplicates, or were not focused on endometriosis. Subsequently, of the remaining 185 articles, 172 were discarded because they focused on elucidating the pathophysiological role of miRNAs (*n* = 23) or used alternative samples, e.g., FFPE or saliva, *n* = 3 (Figure 1).

From among the relevant studies, miRNAs that had shown the greatest diagnostic potential and that had been previously evaluated in Hispanic populations were selected. Thus, the haplotype of this study included hsa-miR-125b-5p (MIMAT0000423), hsa-miR-150-5p (MIMAT0000451), hsa-miR-451a (MIMAT0001631), hsa-miR-3613-5p (MIMAT0017990), hsa-miR-342-5p (MIMAT0004694), and hsa-let-7b-5p (MIMAT0000063) (Table 1).

Subsequently, a total of 84 blood samples were collected from patients with clinical suspicion of endometriosis, all of whom underwent laparoscopic surgery following sample collection. Sample quality was assessed based on the absence of hemolysis, successful amplification of exogenous RNA (UniSP6), detection of the endogenous control gene (snRNA U6), and availability of a confirmed diagnosis. During plasma isolation and initial quality screening, visibly hemolyzed samples were excluded, yielding 60 suitable samples. These were processed for miRNA purification and reverse transcription, with UniSP6 (Ct = 20 ± 2) serving as an exogenous quality control. Three additional samples were excluded due to failure in U6 amplification, resulting in 57 high-quality samples. Finally, after double-blind diagnostic confirmation via laparoscopy, a subset of samples was further excluded due to unresolved quality issues, leaving 22 samples for analysis: 15 from patients with confirmed endometriosis (stages I, II, and IV according to the rASRM classification), and 7 from the reference group (Table 2).

**Table 2:** Available demographic and clinical characteristics of study participants.

Variable	Endometriosis group (*n* = 15)	Reference group (*n* = 7)
Mean age (years) ± SD	38.3 ± 9.4	36.9 ± 7.1
Stage I	9 (60.0%)	0 (0.0%)
Stage II	5 (26.6%)	0 (0.0%)
Stage III	0 (0.0%)	0 (0.0%)
Stage IV	3 (13.4%)	0 (0.0%)
Surgical procedure	Diagnostic/Operative laparoscopy	Total laparoscopic hysterectomy (*n* = 6); Myomectomy (*n* = 1)
Diagnostic confirmation	Endometriosis by laparoscopic visualization and staging (rASRM)	Surgery performed for benign gynecologic indications; endometriosis not reported^a^

^a^ Reference group is reported by procedure (total laparoscopic hysterectomy or myomectomy). No laparoscopic or histopathological evidence of endometriosis status was observed for this group.

The demographic characteristics revealed that the mean age of patients with endometriosis was 38.3 ± 9.4 years, while the reference group presented a mean age of 36.9 ± 7.1 years. In the endometriosis group, 60% were diagnosed at stage I, 26.6% at stage II, and 13.4% at stage IV; no stage III cases were identified. In the reference group, all participants underwent total laparoscopic hysterectomy for benign gynecological conditions. Data regarding body mass index, menstrual cycle phase, and hormonal medication use were not available for all participants. Menstrual cycle information was not accurately recorded due to irregularities and hormonal treatments; however, previous studies have reported that the expression of the miRNAs included in this study is independent of the menstrual cycle phase, BMI, and age (8).

**Figure 2:**
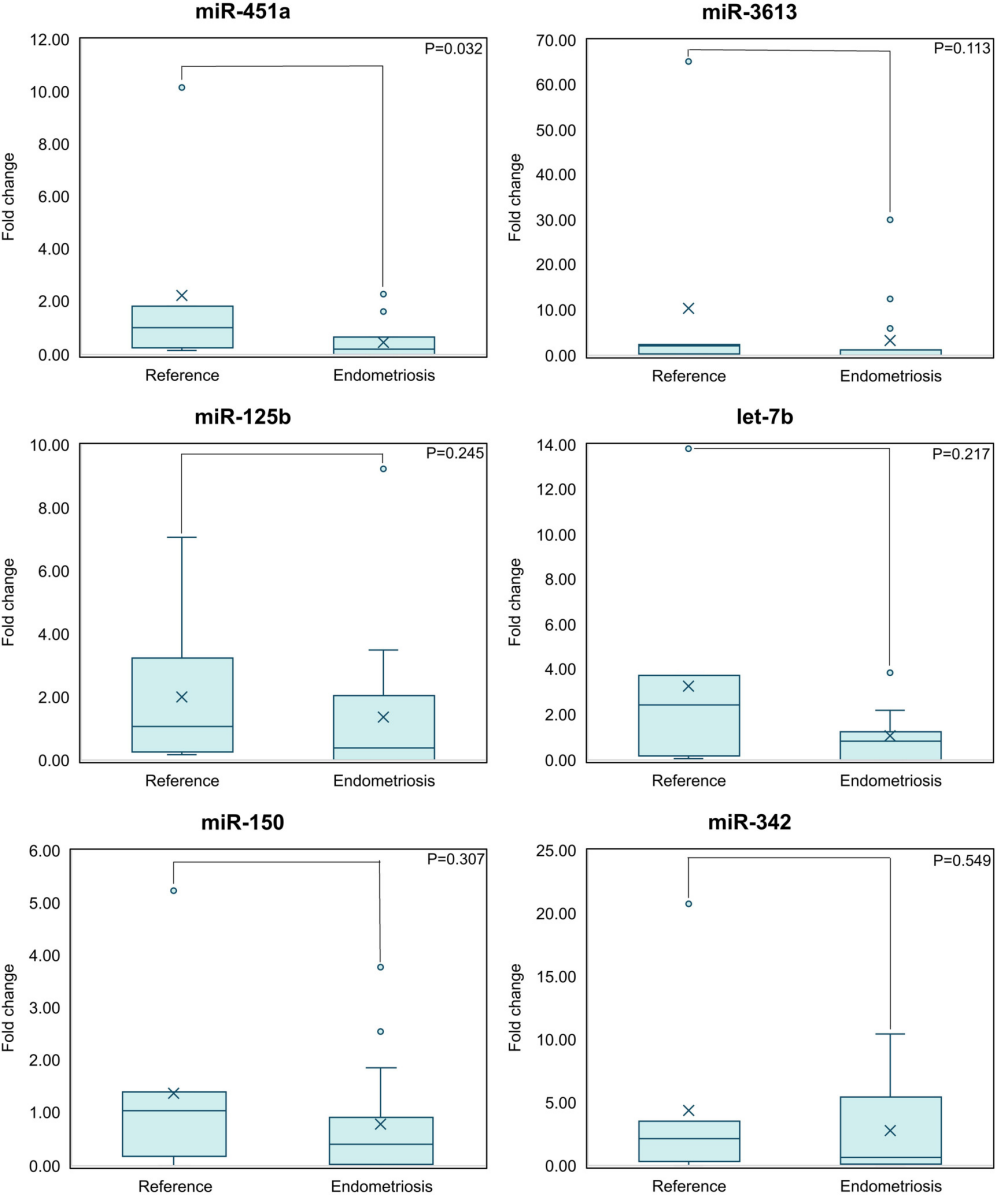
miR-451a has the potential to be a biomarker for endometriosis by itself; is the only miRNA with a statistically significant difference (*P* < 0.05) as compared to the reference group. Means comparison between the reference and endometriosis groups is shown (Mann–Whitney *U*-test).

The quantification of the relative expression of the haplotype miRNAs was performed double-blinded by RT-qPCR, using the U6 gene as a constitutive control. Normality tests (Shapiro–Wilk, *P* ≤ 0.05) indicated that the data did not follow a normal distribution, so the nonparametric Mann–Whitney *U*-test was used to compare the differences between groups (Figure 2). This analysis showed that, of the miRNAs evaluated, only miR-451a was significantly downregulated in patients with endometriosis (*P* = 0.032). Although the rest of the miRNAs did not reach statistical significance, a trend toward decreased expression was observed in the endometriosis group.

**Figure 3:**
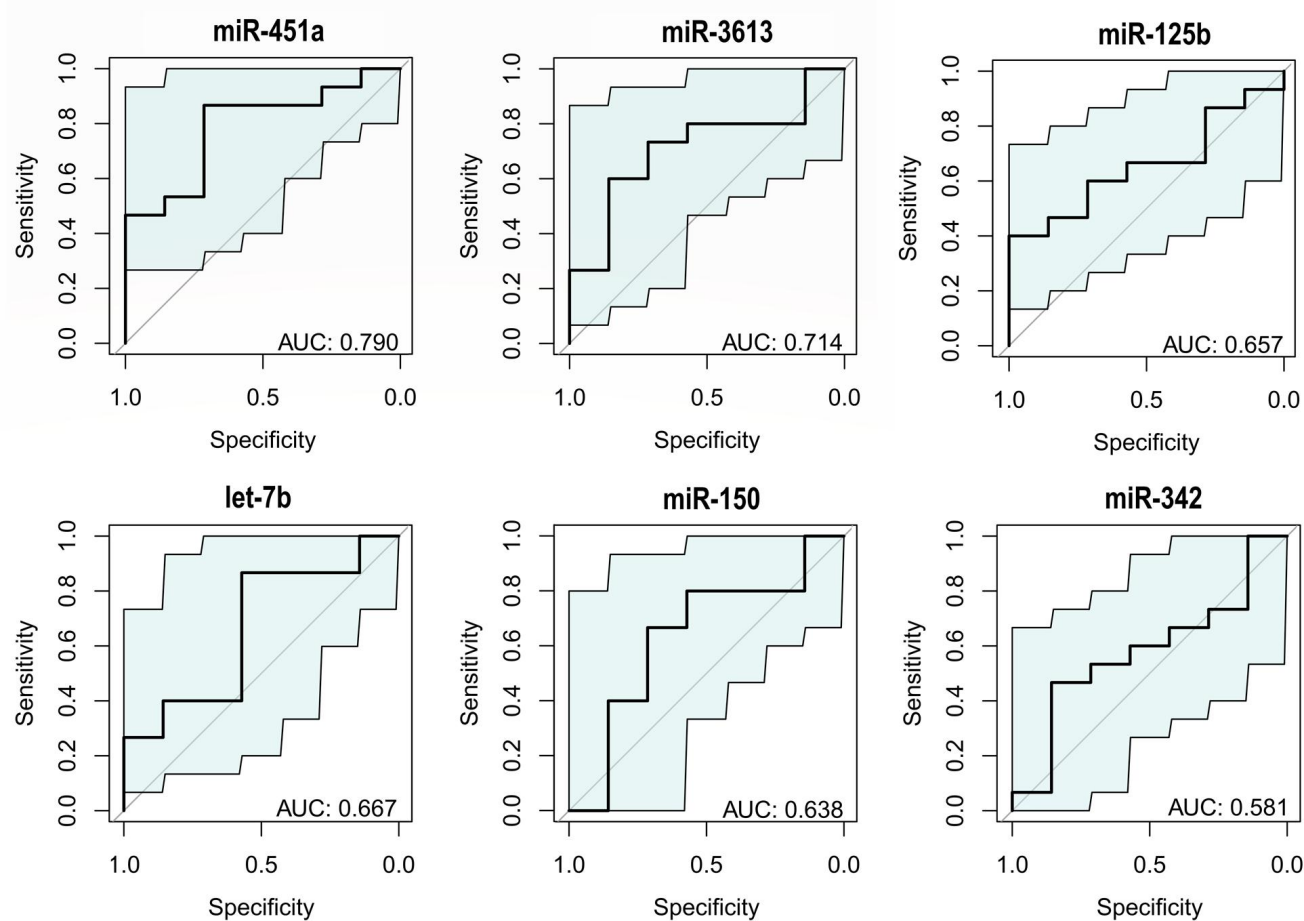
miR-451a exhibits the highest discriminative power (AUC = 0.790). Individual ROC curves of miRNAs performed by pROC package in R Studio (18, 19).

Given that the comparison of sample means is limited in non-parametric data and with a small sample size, it was considered of greater relevance to evaluate the discriminative power of each miRNA using individual ROC curves (Figure 3). In this analysis, the AUC ranged from 0.581 (miR-342) to 0.790 (miR-451a). Individual sensitivities ranged from 40% (miR-125b) to 86.6% (miR-451a and let-7b), while specificity ranged from 57.1% (let-7b and miR-150) to 100% (miR-125b). Considering all metrics (Table 3), miR-451a had the highest individual diagnostic power, followed by miR-3613 and let-7b.

**Table 3:** Metrics obtained by individual ROC analysis of miRNAs performed by pROC package in R Studio (18, 19).

miRNA	AUC	Sensitivity (%)	Specificity (%)	Optimal cutoff	95% CI
miR-451a	0.790	86.7	71.4	0.68	0.59–0.99
miR-3613	0.714	73.3	71.4	0.05	0.48–0.95
miR-125b	0.650	40.0	100	0.04	0.42–0.89
let-7b	0.667	86.7	57.1	2.22	0.40–0.93
miR-150	0.638	80.0	57.1	0.56	0.35–0.92
miR-342	0.586	46.7	85.7	0.33	0.31–0.85

**Figure 4:**
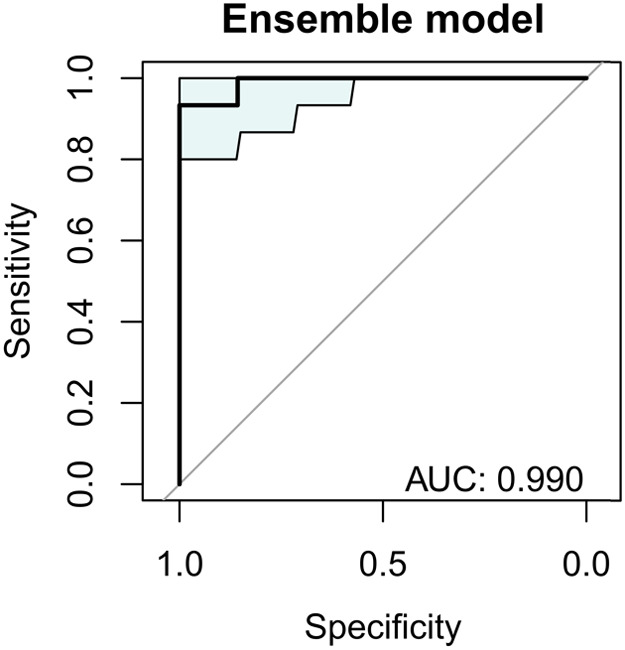
Expression haplotype of miRNAs (miR-451a, miR-3613, let-7b, miR-342, miR-125b, and miR-155) showed a high discriminative capacity (AUC = 0.990) between Hispanic individuals with and without endometriosis. ROC curve corresponds to a stacking-based ensemble model combining Logistic Regression and Classification and Regression Tree (CRT) analyses.

The integration of classification algorithms, using logistic regression and CRT models, allowed the evaluation of the diagnostic capacity of the set of 6 miRNAs. In the logistic regression model, the incorporation of miRNAs as predictor variables improved the classification of the sample, reaching an overall percentage of 81.8% and a high discrimination power (AUC = 0.914; sensitivity 80%; specificity 100%) (Table 4), being the most significant variables in the equation miR-451a (*P* = 0.176), let-7b (*P* = 0.175), and miR-3613 (*P* = 0.219). In parallel, the CRT analysis generated a decision algorithm at three levels, in which miR-451a (improvement = 0.146), miR-3613 (improvement = 0.069), and miR-let-7b (improvement = 0.121) stood out, achieving a correct classification of 95.5% (cross-validation was performed; AUC = 0.990; sensitivity 100%; specificity 85.7%). In addition, a stacking ensemble model was implemented that combined both algorithms, obtaining a discriminative capacity of AUC = 0.990, with a sensitivity of 93.3%, and a specificity of 100% (Figure 4).

**Table 4:** Accuracy of binary classification models to assess the diagnostic capacity of miRNAs in Hispanic patients with and without endometriosis.

Model	AUC	Sensitivity (%)	Specificity (%)	95% CI
Logistic Regression	0.914	80.0	100	0.79–1
CRT	0.990	100	85.7	0.97–1
Ensemble model	0.990	93.3	100	0.96–1

## 4 Discussion

In this study, the discriminative capacity of a miRNAs expression haplotype to differentiate between Hispanic women with and without endometriosis was evaluated. Up to our knowledge, this is the first study specifically targeting this population with miRNAs approach. The results show that the expression haplotype, composed of six miRNAs and analyzed using a stacking-based ensemble model, which integrates logistic regression and classification trees (CRT), reached an AUC of 0.990, with a specificity of 100% and a sensitivity of 93.3%. These findings indicate a high robustness and reproducibility of the model, suggesting its potential as a complementary tool in the diagnosis of endometriosis before laparoscopy in Hispanic patients. This could represent a non-invasive alternative with relevant clinical implications, allowing an earlier diagnosis, optimizing the management of the disease and reducing its socioeconomic impact.

So far, most studies on miRNA expression in endometriosis have been conducted in North American, European, and Asian populations, limiting the generalizability of their findings to other ethnic groups. For example, a study conducted in an Iranian population in 2025 demonstrated moderate to acceptable diagnostic accuracy for endometriosis based on differential expression of eleven miRNAs, with miR-340 showing a particularly high diagnostic value (AUC = 0.846) (20). Another study, conducted in a Chinese population, reported that a panel comprising miR-199a, miR-122, miR-145, and miR-141, combined with serum CA125 levels, achieved an AUC of 0.939 for diagnosing endometriosis (21). To date, the only study that has included Hispanic patients—though in a limited subset—was conducted by Moustafa et al. (8). Therefore, the present study represents a significant advancement in biomarker research for endometriosis and helps fill a critical gap in the literature by providing population-specific data for Hispanic women, a group historically underrepresented in miRNA expression studies. This is particularly important, as genetic and epigenetic differences between populations can influence biomarker expression, underscoring the need to develop or validate diagnostic tools tailored to specific population groups (11).

Currently, there are several strategies for the diagnosis of endometriosis in patients with suspected disease, with diagnostic powers ranging from 82% to 98.4%. These include the implementation of questionnaires and symptomatology classification (22), imaging techniques such as transvaginal ultrasound (23), and magnetic resonance imaging (24), as well as biomarkers such as CA125, IL-6, glycodelin, IGFBP-3, and VEGF (25). In recent years, additional circulating biomarkers, including specific miRNA and piRNA signatures (21, 26, 27), extracellular vesicle profiles (28, 29), and metabolomic patterns (30) have been investigated, showing promising but variable performance across different populations. Moreover, bioinformatic approaches, such as machine learning and artificial intelligence, have been explored (31), as well as genomic and transcriptomic analyses (10). However, many of these strategies may not be suitable for the Hispanic population due to limitations in accuracy, sensitivity, or specificity, as well as economic and operational barriers. Therefore, the implementation of a diagnostic method with high specificity and sensitivity, based on a defined miRNA expression haplotype validated in the Hispanic population, represents a viable and potentially scalable alternative, particularly since it relies on precise and accessible molecular biology techniques, such as RT-qPCR.

An advantage of the expression haplotype including miR-451a, miR-3613, miR-125b, let-7b, miR-150, and miR-342, is that previous studies have shown that their expression does not vary significantly with menstrual cycle progression, contraceptive use, or body mass index (8). In this study, a general trend towards the downregulation of miRNAs was observed in patients with endometriosis; however, only miR-451a showed a statistically significant decrease (*P* = 0.032). Regarding individual discriminative capacity, the miRNAs analyzed presented AUC values between 0.581 and 0.790, with miR-451a (AUC = 0.790), miR-3613 (AUC = 0.714) and let-7b (AUC = 0.667) standing out as the most relevant.

miR-451a has previously been reported with up to 10-fold overexpression as fold change, in patients with endometriosis (8, 32). However, other studies with a similar approach discarded miR-451a as a potential biomarker, since, despite its high discriminating power (AUC = 0.828), it was downregulated in patients with endometriosis, contrary to what was previously reported in other populations (33), but that data is consistent with the results obtained in this study. This discrepancy highlights the need for further exploration of this specific miRNA, as factors unrelated to the disease could influence its expression levels. A relevant example is hemolysis in samples, not our case because we discarded those, since miR-451a is predominantly expressed in erythrocytes, which could alter its detection in plasma or serum in the presence of hemolysis (28).

In contrast, miR-3613 and let-7b showed a behavior consistent with previous studies, presenting significant downregulation in patients with endometriosis (8, 28, 33). In addition, the discriminative capacity of these three miRNAs (miR-451a, miR-3613 and let-7b) is consistent with previous studies, and their greatest contribution was observed in our analysis of the complete expression haplotype by logistic regression and CRT. In both models, these three miRNAs showed a great influence, which reinforces their importance in the development of a diagnostic panel for endometriosis.

**Figure 5:**
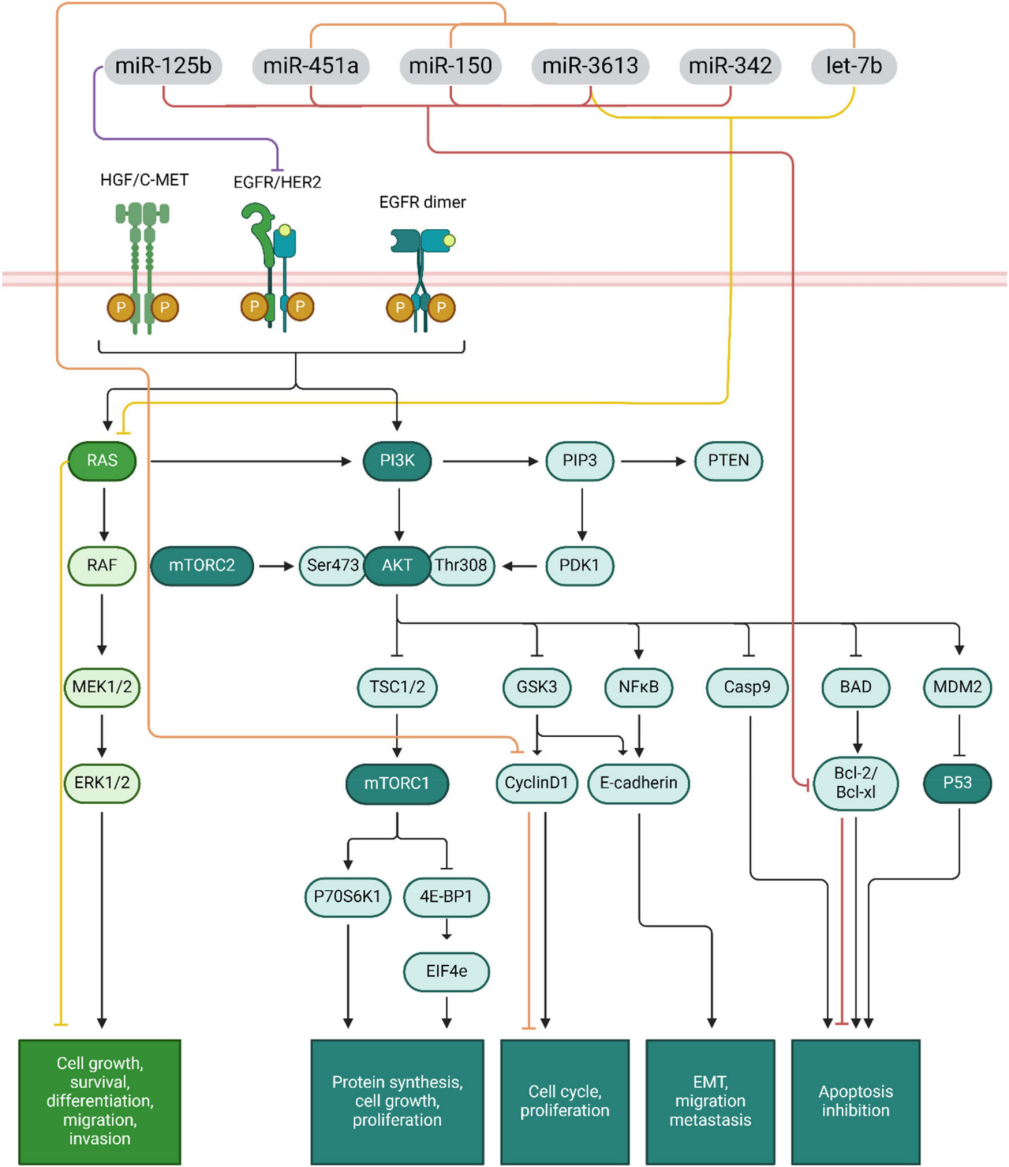
Signaling pathways and functions of the miRNAs included in the expression haplotype evaluated for endometriosis diagnosis, where their participation in processes such as cell cycle regulation, proliferation, survival, and apoptosis stand out. Diagram modified from Lei et al. (34) using Biorender, miRNAs targets verified with miRPathDB, KEGG and TargetMiner.

Bioinformatics miRNA target prediction tools, such as miRPathDB, TargetMiner and KEGG, have demonstrated the involvement of these miRNAs in the regulation of key physiological pathways, including cell proliferation, cell cycle regulation, metastasis, angiogenesis, and hormone regulation, among others. Although endometriosis is not considered a disease with malignant behavior, it shares biological characteristics with neoplasms, which suggests a similar pathogenic mechanism in certain aspects, which is why some of the pathophysiological routes of cancer were taken as a reference (Figure 5).

When analyzing the potential targets of miRNAs, their participation in the negative regulation of genes such as Bcl-2, cyclin D, RAS, and EGFR were identified, which act as proto-oncogenes by promoting cell proliferation (34). Under physiological conditions, the proper expression of these miRNAs maintains control of the cell cycle, preventing uncontrolled growth. However, the downregulation of the miRNAs observed in this, and other studies avoids the negative regulation of these key genes and could be related to atypical cell proliferation, ectopic implantation of endometrial tissue, and metastasis-like features in endometriosis (Figure 5).

The results of this study are promising in the development of a non-invasive diagnostic method for endometriosis in the Hispanic population, based on molecular biology techniques combined with advanced classification models (logistic regression, CRT, and stacking). These methodologies have been optimized for the analysis of a short sample, which represents an advance in the research of biomarkers for this disease. In a potential clinical pathway, this haplotype-based assay could be implemented as an initial screening tool in primary or gynecological care settings for women with suggestive symptoms, thereby guiding referrals for imaging or surgical confirmation only in high-probability cases. Such integration could shorten diagnostic delays, reduce the need for invasive procedures, and optimize healthcare resources. Further large-scale validation will be essential before formal incorporation into diagnostic guidelines.

However, the study has certain limitations, with the sample size being the most relevant. In addition, there is a possible sample selection bias, given that all patients come from the same region. An additional limitation is the partial availability of demographic and clinical data (e.g., BMI, menstrual cycle phase, hormonal medication use), inherent to translational medicine studies. Nevertheless, previous reports indicate that these variables are unlikely to influence the analyzed miRNAs (35, 36). Another consideration is the inclusion of women with other gynecological pathologies in the reference group could generate variability in the results. However, laparoscopy is the only confirmatory method for both the positive and negative diagnosis of endometriosis and clinicians must differentiate patients with and without the disease based on clinical criteria. Thus, including a reference group with other gynecological conditions was considered an appropriate approach. It is also possible that interlaboratory variability in miRNA quantification represents an additional source of bias. It is important to note that validation of these findings in diverse hispanic subpopulations would require a multicenter, collaborative effort, integrating patients from different regions to ensure representativeness. While such a study would be logistically demanding, it remains a crucial step to confirm the generalizability of the diagnostic model. These aspects highlight the need to validate findings in larger cohorts to assess the reproducibility of the diagnostic model. In addition, to better understand the expression levels of the miRNAs analyzed and their biological relevance, it is essential to perform functional studies that confirm their participation in the pathophysiology of endometriosis.

In summary, the miRNAs expression haplotype identified in this study, particularly miR-451a, miR-3613, and let-7b; showed a high discriminating power between patients with endometriosis vs. other pelvic pain but without endometriosis in the studied population (AUC = 0.990). These results reinforce the idea that a diagnostic signature based on multiple miRNAs is more effective than the analysis of single markers. However, its validation in other cohorts is essential before considering its clinical implementation.

## 5 Conclusions

The miRNA expression haplotype composed of miR-451a, miR-3613, miR-125b, let-7b, miR-150, and miR-342: showed a high capacity for discrimination between patients with and without endometriosis in the Hispanic population. The advanced classification models used in this study confirmed their performance: logistic regression (AUC = 0.914), CRT (AUC = 0.990), and stacking-based ensemble model (AUC = 0.990). These findings support its potential use as a diagnostic tool, future validation in a larger cohort must be performed before clinical trials.

## Data Availability

The raw data supporting the conclusions of this article will be made available by the authors, without undue reservation.
